# Supplementary material for: ARA: a flexible pipeline for automated exploration of NCBI SRA datasets
Source: Gigascience. 2023 Aug 17;12:giad067. doi: 10.1093/gigascience/giad067 (PMC10433097; doi:10.1093/gigascience/giad067)
Supplement: giad067_GIGA-D-23-00049_Revision_1 [file giad067_giga-d-23-00049_revision_1.pdf]

GigaScience

ARA: A flexible pipeline for automated exploration of NCBI SRA datasets

--Manuscript Draft--

**Manuscript Number:**	GIGA-D-23-00049R1
**Full Title:**	ARA: A flexible pipeline for automated exploration of NCBI SRA datasets
**Article Type:**	Technical Note
**Funding Information:**	Narodowe Centrum Nauki (2017/27/B/NZ2/00467) — Prof. Wojciech Karlowski
**Abstract:**	Background: One of the most effective and useful methods to explore the content of biological databases is searching with nucleotide or protein sequences as a query. However, especially in the case of nucleic acids, due to the large volume of data generated by the Next Generation Sequencing technologies, this approach is often not available. The hierarchical organization of the NGS records is primarily designed for browsing or text-based searches of the information provided in metadata-related keywords, limiting the efficiency of database exploration.<br>Findings: We developed an automated pipeline that incorporates the well-established NGS data processing tools and procedures to allow easy and effective sampling of the NCBI SRA database records. Given a file with query nucleotide sequences, our tool estimates the matching content of SRA accessions by probing only a user-defined fraction of a record’s sequences. Based on the selected parameters, it allows performing a full mapping experiment with records that meet the required criteria. The pipeline is designed to be easy to operate — it offers a fully automatic setup procedure and is fixed on tested supporting tools. The modular design and implemented usage modes allow a user to scale up the analyses into complex computational infrastructure.<br>Conclusions: We present an easy-to-operate and automated tool that expands the way a user can access and explore the information contained within the records deposited in the NCBI SRA database.
**Corresponding Author:**	Wojciech Karlowski<br>Adam Mickiewicz University in Poznan<br>Poznan, POLAND
**Corresponding Author Secondary Information:**	
**Corresponding Author's Institution:**	Adam Mickiewicz University in Poznan
**Corresponding Author's Secondary Institution:**	
**First Author:**	Anand Maurya
**First Author Secondary Information:**	
**Order of Authors:**	Anand Maurya
	Maciej Szymanski
	Wojciech Karlowski
**Order of Authors Secondary Information:**	
**Response to Reviewers:**	Dear Prof. Nogoy,<br><br>Thank you very much for considering our article entitled “ARA: A flexible pipeline for automated exploration of NCBI SRA datasets” for publication in GigaScience. Your opinion and the comments of the Reviewers have stimulated a number of changes to the manuscript that we think have further improved the article.<br><br>In the revised version of the manuscript and the responses, we have addressed all the issues raised by the Reviewers. In summary, we have investigated the influence of the screening sample size on the false discovery rate of the predictions and introduced several modifications to the documentation and manuscript providing more information

related to the used tools and computational workflow.

All changes to the manuscript are indicated in the text with red color.

Below are our point-by-point responses (A) to all the reviewers' comments (C).

Sincerely,

Wojciech Karlowski

Responses to Reviewers' comments:

Reviewer #1: This package runs a pipeline that handles many of the tasks that a user might want to perform on SRA sequences, including data retrieval, data quality checks, clustering redundant reads, etc. I think the most interesting feature is the ability to search a subset of an SRA run in order to judge it's relevance to a particular problem.

We would like to thank the Reviewer for carefully reading our manuscript and providing comments that address problems related to the presented work.

C: I have some comments/requests.

I think the authors need to provide more details about the screening feature:

In screening mode, 10% of the sequences from a record are downloaded and aligned (by default). I assume this is the first 10% of the reads, as opposed to every 10th read. The authors should state this explicitly. Also, a comment on whether or not looking at only the first 10% might be a non-representative sample would be good.

A: The reads sampling functionality is provided by the fastq-dump program (SRA-toolkit) and according to documentation it fetches the first 10% of the reads. We now provide this information in the manuscript text.

C: I'm confused about what's being shown in suppl. figures 1 and 2. I think these are alignments for all the sequences in the record, though I'm not 100% sure. I think the authors would want to show a comparison of the prediction that results from sampling 10% of the reads and the full run. That is, can they demonstrate, even anecdotally, that this feature works and is valuable? This is important for readers to take the article seriously.

A: These two figures are provided in the manuscript to illustrate the efficiency (sensitivity and computational effectiveness) of two implemented in the ARA pipeline sequence comparison tools (Bowtie2 and BLASTn). Our intention was to present significant differences in the output results related to the used tool (expected) and tested sample (unexpected). We have slightly modified the caption of these figures to state our intentions more clearly.
The current version of the manuscript is also supplemented with the FDR analysis of the case study data set. In a new supplementary data file, we provide detailed information about the efficiency of the sequence comparison tools in a spectrum of screening sampling thresholds, including the full dataset.

C: What is the tRNA database that is used to screen the records for the two suppl figures? It seems that should be available to readers and listed in the Data Availability section.

A: The tRNA sequences are sourced from the official annotation of the Arabidopsis genome located in GtRNAdb database (Arabidopsis thaliana - TAIR10). We have added information to the manuscript stating the source of the reference sequences in the application example.

C: How were the 100 SRA runs for the suppl. figures selected? Some were submitted by the last author (which I have no issue with), but I think it's important that the readers

Powered by Editorial Manager® and ProduXion Manager® from Aries Systems Corporation

know the basis for the selection. Were they part of another project in the lab, were they chosen based on some other criteria that made them interesting, etc.?

A: The ARA pipeline is currently employed in a number of high-throughput screening projects in our lab. One of them deals with the expression of tRNA genes in Arabidopsis thaliana and consists of experimental as well as computational analyses. Hence, some of the samples that appeared on our test screening results are authored by us. This actual finding we consider as a positive control - we have expected that these samples should appear in the results. The original experiment involved screening more than 20 000 SRA accessions. For the presentation of differences between Bowtie2 and BLASTn screening, we have selected from these results representative examples to show the effect on the fragment identification efficiency and computational cost. Hence, these results should not be considered as a benchmark, but rather as a list of representative examples.

C: There are issues with the references. References 2 and 9 are the same. 7 and 10 are also the same, etc.

A: We are sorry for these errors. We corrected and manually verified the references in the revised version of the document.

Reviewer #2: The ARA tool is a very interesting gate for the preliminary exploration of a target sequence to the massive SRA repository. Using ARA, the user is able to more efficiently mine samples of relevance from SRA, as well as have initial insights on the spread of your target sequence. In addition, the tool's modularity should increase its usability. I particularly like the "both" exploration method. For these reasons, I recommend the publication of the ARA pipeline with some reviews. Congratulations on your work.

We would like to thank the Reviewer for positive feedback on our work.

Major Reviews:

C: The tool is an easy-to-use approach for preliminary sequence exploration. However, it is not novel. Although its user-friendliness is a selling point, stricter validation should be provided for the screening process.

A: We do agree that the concept of preliminary sequence screening is not novel. However, to our best knowledge, it was never implemented in the case of huge NGS sequence resources. Additionally, our approach is very flexible - it allows switching sequence comparison programs (currently two implemented) and an easy expansion of the available toolbox by almost any additional tool. We provide in the introduction information about other tools that employ similar strategies and compare their functionalities in Table 1.

C: The screening approach downloads, by default, 10% of the sequence file from SRA to use with the query sequence. Although the authors allow the user to change the default value, no value is given as a recommendation, and no explanation is provided as to why 10% was chosen as the default cut-off.

A: We do agree that it is very difficult to establish one fixed parameter representing effectively the content of any sequence record. The suggested 10% threshold was mainly based on our practical observation - the value that was most useful in a wide spectrum of records and applications. Additionally, some of the recent studies (e.g., https://doi.org/10.1186/s12859-022-04572-7 and https://doi.org/10.1016/j.xhgg.2022.100103) demonstrate that 10% is a representative fraction of an NGS sequencing record.
However, based on the suggested by the Reviewer FDR analysis, in the updated version of the pipeline, we set as default the 5% value. For more explanation please see our answer below.

C: What exploratory bias would a user have if they only used the screening exploration method? What is the False Negative rate of the screening method, i.e., how often I won't find my sequence in an SRA sample simply by chance?

A: This is a very hard question to answer because the value will depend on the query sequence set, the inclusion criteria (positive or negative classification of a sample based on the percent of matching fragments), and the selection of the samples. However, following the Reviewer's advice, we have performed the calculations on our test data set (207 SRA samples tested with tRNA transcripts from Arabidopsis thaliana) to gain a preliminary glimpse into the usefulness of the screening procedure. The results are now provided as a new Supplementary File 1 and show that the 5% threshold (lowest) provides good estimates at the FDR of 0.0727. Only 4 NGS runs (from experiment SRP267192) required a higher screening threshold value and behaved erratically during the analysis (for details please see Supplementary File 1).

C: I would like to see a recommendation for the cut-off percentage based on an optimal balance of False Negative and Storage efficiency exploration.

A: As stated in the previous answer, it may be impossible to assess a universal cut-off value that will represent all possible research scenarios (reference sequences and SRA records' characteristics). Nevertheless, with further exploration of our tRNA example, we show the effect of the screening threshold value and provide the results in Supplementary File 1. From these analyses, we conclude that the lowest tested value (5%) is sufficient in most cases to successfully select the sample. However, even in this example, we have detected erratically behaving samples (SRR12008191-4). Therefore, the proposed by us default value should be considered merely as a suggestion and should be treated as a starting point for further exploration by a pipeline user.

C: In addition, how is your sampling done? Do you randomly download 10% of the sequences, or is there some sort of sampling pattern?

A: The sequence files hosted on NCBI SRA are submitted by the researchers. Based on their original order in the supplied file, we download the data using '-X' option in fastq-dump tool. It's vital to note that the submitter may have sorted, aligned, transformed, or generated the fastq file randomly before uploading. To truly randomize the record content we would need to download the whole dataset, which is in obvious contrast with the idea of fast screening of the records.

C: In line 157, the authors mention downloading a "representative" fraction of the sequences from each record. However, there is no representative analysis shown.

A: We agree that this statement was misleading. We have corrected the sentence by removing the 'representative' adjective.

Minor Reviews:

C: Line 74 (… development of specialized solutions (e.g., BOWTIE [3] and BWA [4]) that can process sequence data more efficiently than the classical methods):

It is not clear what are the classical methods. Are you referring to FASTA and BLAST? Please make it more transparent. In addition, please provide a reference or metric that indicates the comparison of efficiency among the methods.

A: We are referring here to classical database exploration tools like BLAST and FASTA (introduced in the previous paragraph). We have modified the sentence to make it clear. We also explain in this sentence the mentioned efficiency metrics as the required computational time.

C: Line 80 (… This is an obvious limitation since the user must depend on the completeness and correctness of the information provided in record descriptions.):

Powered by Editorial Manager® and ProduXion Manager® from Aries Systems Corporation

Can you provide proof or indicate a reference that shows that depending on the records metadata is, in fact, a limitation?

A: There are instances where the information about the run in the SRA repository, as well as the matching sample-level attributes in the BioSample repository, is missing or incomplete. For example, the SRA accessions SRR499990, ERR2596563, and ERR1769645. If the metadata is inconsistent or incomplete, finding samples that contain sequences of interest might be difficult in NCBI SRA.

C: Line 119 (…In such a case, the files are retrieved using the wget program, and the NGS reads are extracted with fastq-dump. However, the fastq-dump supports downloading of a fraction of reads from a particular sequencing run accession.):

I don't understand the use of adversity in the sentence, i.e., 'However.' If I understand correctly, the ability to download only partial sequence is by design.

A: The adversity was placed here to contrast the functionality of fastq-dump and wget downloading modes. Following the Reviewer's suggestion, we have removed it from the sentence.

C: Line 138 (… . By default, the tool uses a viral genomic reference dataset.):

Is there a particular reason why you choose this as default? Please provide a sentence justifying the selection of this default.

A: We are using the viral genomic database (release: 9/8/2022) because it's smaller in size and quicker to set up during the installation of the pipeline. A user can download a larger reference and use it in the pipeline after the installation is finished.

C: Line 142 (The whole Installation description)

Please provide the versions of all used tools as supplementary material. I understand that most of the tool's version can be found in the requirements file for the conda installation. However, it is relevant to reproducibility to provide the version of all used tools and dependencies clearly.

The manuscript installation section also does not clearly state the amount of computer resources needed to install ARA. Please provide the storage requirements for the tool itself as well as an estimation of how much extra storage is required after ARA generates the results.

A: Since the version of the used tools and required space for installation may change frequently, we provide now this information in the pipeline documentation.

C: Line 170, figure 1 legend (… Blue arrows indicate steps executed in "full' mode. Red arrows show the analysis path specific for the combined "screen + full" mode)

Instead of "screen + full" please use "both" as this was the name of the method previously introduced on line 160.

A: Thank you for spotting this oversight. We have corrected the figure caption accordingly.

C: Line 198, Table 1:

Please include in the table which of the tools is able to do partial data download. Or do you imply this in the "data screening mode"?

A: The ability to download partial data is referred to as "data screening mode". The table has been modified to improve clarity.

C: The magicblast tool is not compared with the ARA tool in the introduction. Please do

so.

A: We now include a short description of MagicBlast in the introduction.

C: Line 206 (… Upon completion of the analysis, the pipeline also generates a combined summary file sorted by the overall alignment percentage in decreasing order (Supplementary Table 1))

Please indicate, somehow, on the final results file which method ARA used at the end and that the rows are sorted.

A: The final table is sorted in decreasing order of the total percentage of alignment. It measures how many reads out of the total in the sample have been aligned by the aligner (BLASTn/BOWTIE2). Additionally, we now indicate the tool as well as the sorting status in the name of the output file.

C: Line 229 (A case study…):

Please include a comparison to the currently used approaches. How much more efficient is your screen mode? What would be the result of your exploration using the classical methods?

A: Unfortunately, none of the currently available methods that can process automatically SRA records does provide a 'screening' mode (please see Table 1). However, since all the other tools download the full record data and the mapping algorithms are common between them, we may assume that, on the same computational architecture, their time performance would be worse.

C: Line 255 (Programming language: Perl):

Please provide the version.

A: We provide now this information in the 'Availability and Requirements' section.

GitHub page and Installation procedure:

Installation:

C: Please provide detailed instructions on downloading and extracting the zip file. Which zip file? If I understand, the more straightforward approach would be to use 'git clone' or download the complete repository as a zip using 'wget'.

When using git clone, the instructions should change to cd ARA-main
'''original
cd ARA/
mamba env create --file requirements.yaml
mamba activate ara_env
perl setup.pl
´´´
'''edited
cd ARA-main/
mamba env create --file requirements.yaml
mamba activate ara_env
perl setup.pl
´´´
A: Thank you for spotting this oversight. We have updated the instructions.

Errors and comments

C: #1
perl ara.pl --help

Powered by Editorial Manager® and ProduXion Manager® from Aries Systems Corporation

perl: warning: Setting locale failed.
perl: warning: Please check that your locale settings:
LANGUAGE = (unset),
LC_ALL = (unset),
LC_CTYPE = "UTF-8",
LANG = "en_US.UTF-8"
are supported and installed on your system.
perl: warning: Falling back to a fallback locale ("en_US.UTF-8").
Can't locate Config/Simple.pm in @INC (you may need to install the Config::Simple module) (@INC contains: /mnt/tools/miniconda3/envs/ara_env/lib/perl5/5.32/site_perl /mnt/tools/miniconda3/envs/ara_env/lib/perl5/site_perl /mnt/tools/miniconda3/envs/ara_env/lib/perl5/5.32/vendor_perl /mnt/tools/miniconda3/envs/ara_env/lib/perl5/vendor_perl /mnt/tools/miniconda3/envs/ara_env/lib/perl5/5.32/core_perl /mnt/tools/miniconda3/envs/ara_env/lib/perl5/core_perl .) at ara.pl line 11.
BEGIN failed--compilation aborted at ara.pl line 11.

#1 Solution
I managed to solve this error by correcting my paths. I suggest you include in the troubleshooting section how to configure your Perl paths:

~/.cpan/CPAN/MyConfig.pm

Specifically, I had problems with my path from 'tar' and 'make.'

A: Thank you for this suggestion. We have created a troubleshooting section to address this and all other issues with the installation, configuration, and execution of the ARA tool.

C: #2

Can't locate Parallel/ForkManager.pm in @INC (you may need to install the Parallel::ForkManager module) (@INC contains: /mnt/tools/miniconda3/envs/ara_env/lib/perl5/5.32/site_perl /mnt/tools/miniconda3/envs/ara_env/lib/perl5/site_perl /mnt/tools/miniconda3/envs/ara_env/lib/perl5/5.32/vendor_perl /mnt/tools/miniconda3/envs/ara_env/lib/perl5/vendor_perl /mnt/tools/miniconda3/envs/ara_env/lib/perl5/5.32/core_perl /mnt/tools/miniconda3/envs/ara_env/lib/perl5/core_perl .) at ara.pl line 12.
BEGIN failed--compilation aborted at ara.pl line 12.

#2 Solution
I could not fix this using only Perl. I fix it by installing the missing dependencies using conda/mamba:

mamba install -c bioconda perl-parallel-forkmanager

A: We have updated the troubleshooting section to include the solution to this configuration problem.

C: #3
If the warning messages are irrelevant, I would suppress them, as the log files become polluted.

A: We have added an option to suppress warning messages in the log.

C: #4

I would like a more detailed description of your output files—for instance, their naming conventions.

A: We have updated documentation to provide a description of the naming conventions used by the ARA tool.

C: #5
If some of your output files are results from other tools, please link the user to the file description from the original tool.

A: We have updated documentation to provide links to the tools implemented in the ARA pipeline.

C: #6
Indicate the storage results. From the example run, a 4MB file generated 592 MB results. An increase of 148 times. I understand that it depends on how many SRA samples the users want to explore, but it would be good to see some sort of estimation of required resources per average size of the reference sample.

A: We provide in the pipeline documentation additional information about storage statistics related to the presented example. This information may offer a user, not familiar with NGS data processing, a preliminary view into the storage needs of a particular project. Unfortunately, we are unable to provide any universal estimation of these values.

C: #7
From the Example run, sample SRR12548227 did not output all folders. Is this expected? Why?

My results were:

screening_results/SRR12548227:
fastQC raw_fastq trimmed_data

screening_results/SRR7289585:
blastn bowtie2 fastQC kraken2 raw_fastq trimmed_data

screening_results/SRR8392720:
blastn bowtie2 fastQC kraken2 raw_fastq trimmed_data

A: We have tried several times to reproduce this error without success. It might be related to the specific configuration of the operating system. We recommend checking the 'runlog.SRR12548227.txt' file generated in the output folder for any error messages. We provide this debugging hint in the troubleshooting section of the documentation.

C: #8
conf.txt file
For tool-specific parameters, please indicate where the user can find information about the parameter

A: We now provide this information in the pipeline documentation.

C: #9
I would like to have ara.pl on the PATH of my ara_env conda environment.
Instruct the user to add ara.pl to the conda PATH

A: We have added this information to the pipeline documentation.

C: #10

I got a relevant error while trying to install using docker. Please, check if the installation is correct. Following is my error:

Err:16 http://archive.ubuntu.com/ubuntu jammy-updates/restricted amd64 Packages

	#0 1038.3 Connection timed out [IP: 185.125.190.39 80]<br>#0 1038.4 Ign:17 http://archive.ubuntu.com/ubuntu jammy-updates/main amd64 Packages<br>#0 1038.4 Fetched 1185 kB in 17min 18s (1141 B/s)<br>#0 1038.4 Reading package lists...<br>#0 1038.4 E: Failed to fetch http://security.ubuntu.com/ubuntu/dists/jammy-security/restricted/binary-amd64/Packages Connection timed out [IP: 185.125.190.39 80]<br>#0 1038.4 E: Failed to fetch http://archive.ubuntu.com/ubuntu/dists/jammy/universe/binary-amd64/Packages Connection timed out [IP: 91.189.91.39 80]<br>#0 1038.4 E: Failed to fetch http://archive.ubuntu.com/ubuntu/dists/jammy-updates/restricted/binary-amd64/Packages Connection timed out [IP: 185.125.190.39 80]<br><br>A: We have checked the Docker image. The above-shown error messages indicate problems with the network connection or server availability. In such a case the installation procedure should be repeated. We have provided a short description of this error in the troubleshooting section of the documentation.<br><br>C: #11<br>Add an outputs section on the GitHub page explaining the expected outputs folder structure and files<br><br>A: We provide the description of the directory structure and file naming convention in the documentation (please see also answer to comment #4).
**Additional Information:**	
**Question**	**Response**
Are you submitting this manuscript to a special series or article collection?	No
**Experimental design and statistics**<br><br>Full details of the experimental design and statistical methods used should be given in the Methods section, as detailed in our Minimum Standards Reporting Checklist. Information essential to interpreting the data presented should be made available in the figure legends.<br><br>Have you included all the information requested in your manuscript?	Yes
**Resources**<br><br>A description of all resources used, including antibodies, cell lines, animals and software tools, with enough information to allow them to be uniquely identified, should be included in the Methods section. Authors are strongly encouraged to cite Research Resource	Yes

Identifiers (RRIDs) for antibodies, model organisms and tools, where possible.<br><br>Have you included the information requested as detailed in our Minimum Standards Reporting Checklist?	
**Availability of data and materials**<br><br>All datasets and code on which the conclusions of the paper rely must be either included in your submission or deposited in publicly available repositories (where available and ethically appropriate), referencing such data using a unique identifier in the references and in the "Availability of Data and Materials" section of your manuscript.<br><br>Have you have met the above requirement as detailed in our Minimum Standards Reporting Checklist?	Yes

Revised manuscript Click here to access/download;Manuscript;ARA manuscript - revision.docx

ARA: A flexible pipeline for automated exploration of NCBI SRA datasets

Anand Maurya

Department of Computational Biology, Institute of Molecular Biology and Biotechnology, Faculty of Biology, Adam Mickiewicz University in Poznan, Uniwersytetu Poznanskiego 6, 61-614 Poznan, Poland

email: anamau@amu.edu.pl

Maciej Szymanski

Department of Computational Biology, Institute of Molecular Biology and Biotechnology, Faculty of Biology, Adam Mickiewicz University in Poznan, Uniwersytetu Poznanskiego 6, 61-614 Poznan, Poland

email: mszyman@amu.edu.pl

Wojciech M Karlowski*

Department of Computational Biology, Institute of Molecular Biology and Biotechnology, Faculty of Biology, Adam Mickiewicz University in Poznan, Uniwersytetu Poznanskiego 6, 61-614 Poznan, Poland

email: wmk@amu.edu.pl

*) corresponding author

Abstract

Background

One of the most effective and useful methods to explore the content of biological databases is searching with nucleotide or protein sequences as a query. However, especially in the case of nucleic acids, due to the large volume of data generated by the Next Generation Sequencing technologies, this approach is often not available. The hierarchical organization of the NGS records is primarily designed for browsing or text-based searches of the information provided in metadata-related keywords, limiting the efficiency of database exploration.

Findings

We developed an automated pipeline that incorporates the well-established NGS data processing tools and procedures to allow easy and effective sampling of the NCBI SRA database records. Given a file with query nucleotide sequences, our tool estimates the matching content of SRA accessions by probing only a user-defined fraction of a record's sequences. Based on the selected parameters, it allows performing a full mapping experiment with records that meet the required criteria. The pipeline is designed to be easy to operate — it offers a fully automatic setup procedure and is fixed on tested supporting tools. The modular design and implemented usage modes allow a user to scale up the analyses into complex computational infrastructure.

Conclusions

We present an easy-to-operate and automated tool that expands the way a user can access and explore the information contained within the records deposited in the NCBI SRA database.

Keywords: SRA database, NGS data, database searching, sequence analysis

Background

The development of new computational tools dedicated to exploring the content of the protein and nucleotide sequence repositories revolutionized the methods of biological data access. FASTA [1] and BLAST [2] algorithms enable fast and efficient search of large databases for similar sequences and a statistical evaluation of the results. The heuristic algorithms used by these tools are much faster than implementations of rigorous dynamic programming algorithms. These methods, however, have a limited application in the context of large data

sets generated by high throughput Next Generation Sequencing (NGS) technologies. To meet the requirements of the NGS data processing, new approaches and specialized tools had to be developed.

The explosion of new techniques and algorithms for NGS sequence mapping led to the development of specialized solutions (e.g., BOWTIE [3] and BWA [4]) that can process sequence data more efficiently (in terms of required computational time) than the classical methods of sequence data exploration (e.g., BLAST and FASTA). However, although more effective, the new programs do not offer an easily accessible way for sequence-based searching of the NGS data repositories (e.g., Sequence Read Archive - SRA [5] or European Nucleotide Archive - ENA [6]). Due to the large volume of data and huge computational time required for sequence searches, exploration of the NGS databases is routinely restricted to text-based surveys with keywords and phrases provided in records' metadata. This is an obvious limitation since the user must depend on the completeness and correctness of the information provided in record descriptions.

The sequence-based screening of the whole SRA database records has been implemented recently in Magic-BLAST tool [7], designed specifically for mapping of large next-generation RNA or DNA sequencing runs. This functionality has been also incorporated, to some extent, into Bowtie2 since release 2.3.5 [8]. The availability of an option to automatically fetch SRA records is reported in prepackaged builds or alternatively, requires the source code compilation using the SRA software dependencies. A similar approach is offered by the *sra-pipeline* tool (https://github.com/FredHutch/sra-pipeline). Here, the program automates the process of downloading, mapping (BOWTIE2 [8]), compressing, and storing the results within the AWS cloud computing infrastructure. More advanced functionality, providing automated access to SRA records, is available in the *pyrpipe* Python package [9]. This tool helps, in a very convenient way, to incorporate the ability for automated RNA-seq data download into a custom pipeline. It also provides a programming interface for tools designed for basic sequence processing applications (e.g., adapter trimming). The *BICF SRA Pipeline* was created with a similar aim, which allows bulk download of data and quality assessment (https://doi.org/10.5281/zenodo.3739788). All of these currently available tools are designed to work by downloading the entire record data (FASTQ formatted files), which in most cases is very expensive in terms of computational resources and storage.

We have developed an integrated, feature-rich, universal, flexible, and ready-to-use solution that allows access to the data using both the SRA-tools and SRA-cloud sources. It provides a full or partial SRA record analysis mode and a choice of the sequence screening method

(BLAST [2] and BOWTIE2 [8]) and taxonomic profiling (Kraken2 [10]). The modular design of the pipeline allows easy further expansion of the sequence analysis toolbox. The implemented procedure also provides basic quality checks, including the removal of adapters and filtering of reads. Along with sequence data processing the pipeline extracts metadata information for each of the SRA records. A simple configuration schema allows full control of the procedure workflow, including the option to resume interrupted analyses and effortless integration into the distributed computational infrastructure.

ARA pipeline workflow

Implementation and tools

Similarly to *Entrez Direct* (command line utilities used to access in a text-based way NCBI databases) [11], the ARA (Automated SRA Records Analysis) tool is implemented in Perl and designed to be used from the shell prompt. It employs the NCBI SRA toolkit (https://github.com/ncbi/sra-tools) to download the raw data in FASTQ format from the SRA database. NCBI Entrez programming utilities provide access to the sample-level metadata along with the location of the raw data stored in the cloud. In case of a problem with *fastq-dump* (SRA toolkit) tool-mediated data downloads, the pipeline is designed to use NCBI e-utilities to find a link to the original SRA file (in the native SRA format) located either at AWS, GCP, or NCBI's storage servers. In such a case, the files are retrieved using the *wget* program and the NGS reads are extracted with *fastq-dump*. The *fastq-dump* supports downloading a fraction of reads from the beginning of a particular sequencing run accession. Using this tool speeds up downloading and analysis processes, which saves time and disk usage. A newer utility, *fasterq-dump*, from SRA toolkit, unfortunately, does not possess all the features offered by *fastq-dump* and is not currently implemented in the ARA pipeline. We will update our software once the developers of SRA toolkit implement the option of partial download in *fasterq-dump*.

Sequence quality evaluation

In order to ensure the highest quality data for processing, the downloaded sequences are subjected to a thorough three-step quality check procedure. FastQC [12] is executed on both raw and adapter-trimmed FASTQ files (**Figure 1** - first and second quality check). Trimmomatic [13] is used to filter low-quality sequences and/or remove adapters, which may profoundly influence the reliability of the alignment and other downstream analyses. The redundant reads are clustered using the Fastx toolkit (http://hannonlab.cshl.edu/fastx_toolkit/)

to reduce the database volume for sequence search and to speed up the analyses. The unique reads are mapped to the query sample using NCBI BLAST command line utility (https://www.ncbi.nlm.nih.gov/books/NBK279690/) and/or BOWTIE2 (**Figure 1**). In addition, the pipeline allows the taxonomic classification of reads using the 'Kraken2' program. By default, the tool uses a viral genomic reference dataset. However, it is possible to use any custom 'Kraken2' database by supplying the required data and modifying the configuration file.

Installation

The pipeline can be effortlessly set up using either Docker or Mamba package manager (https://mamba.readthedocs.io/en/latest/) which is a C++-based implementation of Conda (https://conda.io/). The Mamba implementation offers a faster interface and uses *libsolv* to effectively resolve the dependencies. The use of a package manager guarantees seamless installation of the required software tools along with their dependencies and has been successfully used in many recent bioinformatics projects (e.g., SCRAP [14], *grenepipe* [15], *pyGenomeTracks* [16], *TransposonUltimate* [17], *matOptimize* [18], *plotsr* [19], *CRISPRtracrRNA* [20]. The Perl modules are automatically downloaded and installed by the setup script through CPAN (https://metacpan.org/).

Results and discussion

Workflow modes

The ARA pipeline allows analyses in two major modes: 'screening' and 'full'. Both modes include the same steps (**Figure 1**) that involve basic procedures in NGS sequence data analysis. In the 'screening' mode the ARA tool will perform the analysis only on a small fraction (by default 5%) of sequences from each record. This step is useful in the selection of the best possible candidate datasets for further analyses. The 'full' mode allows downloading and processing of the entire sequence record. The 'both' analysis mode automates the two mentioned steps (first 'screening' and then 'full' analysis) (**Figure 1**). An additional 'summary' mode combines results of independently performed analyses (e.g., on computational grid structure) into one summary file that can be examined and used in subsequent steps (e.g., 'full' mode).

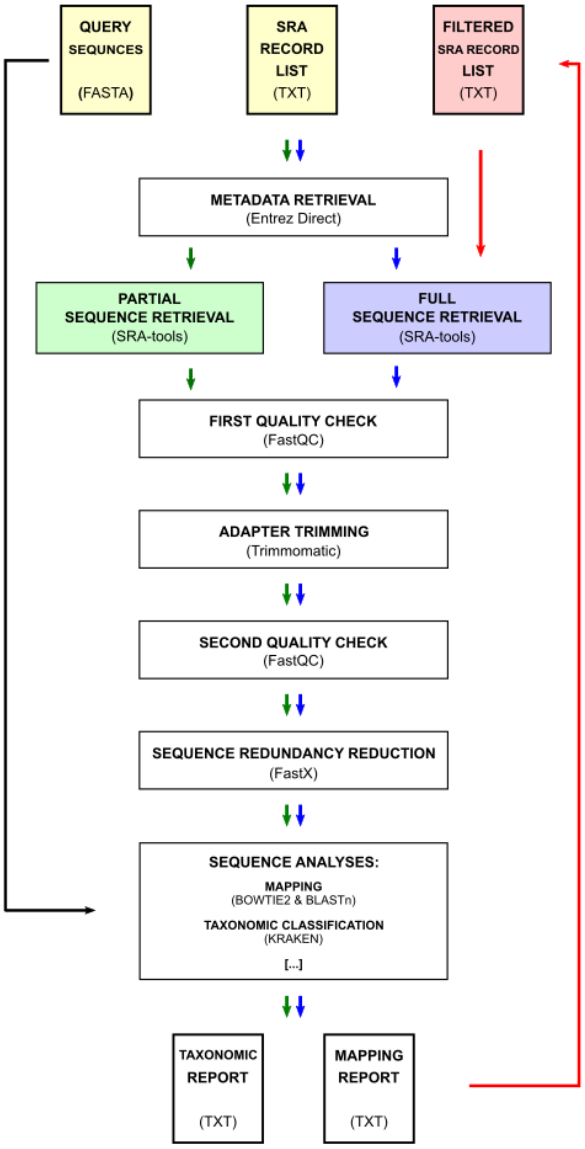

Figure 1. ***Graphic presentation of the ARA pipeline workflow***: *green arrows indicate steps run in the 'screen' mode. Blue arrows indicate steps executed in ''full' mode. Red arrows show the analysis path specific for the combined "both" mode (including automated generation of filtered SRA records list - depicted by a red rectangle). Yellow rectangles indicate user input data (query sequences and list of SRA records' accessions). White rectangles represent distinct steps in the analysis process (with tools indicated in brackets) and output reports generated by*

the pipeline. 'Sequence analyses' currently allow mapping and taxonomic classification, but can be easily expanded to include more tools (depicted by ellipsis in square brackets).

On each SRA 'Run ID' listed in the input file, the ARA pipeline performs data retrieval, adapter trimming, quality check, and mapping of reads using the reference sequences (**Figure 1**).

Features comparison

One of the most important features (**Table 1**) of the ARA pipeline is its modular architecture. Such a design allows an easy expansion of the available toolbox for downstream sequence analyses. In order to demonstrate this functionality, besides sequence mapping tools, we incorporated a module for the taxonomic classification of the reads using Kraken2 [21]. The pipeline generates a classification report for each sample using a provided reference database (viral genomes by default).

In case of problems with downloading the records, the pipeline attempts to retrieve the raw FASTQ file thrice before searching with NCBI e-utilities for alternate SRA file locations in the cloud storage. After successful retrieval of the SRA record, it extracts the single or paired-end reads using the *fastq-dump* utility (SRA toolkit). If both methods to download the data fail, the ARA pipeline logs the event and skips that particular SRA run accession. Following the data download step, the ARA tool performs quality checks to ensure that the absence of matching sequences is not related to sequencing errors. The parameters of each of the analysis steps can be tweaked through the ARA configuration file. A user is required to provide a list of SRA run accessions as the starting parameter for the analysis.

Table 1. *Comparison of the ARA pipeline features with other similar tools.*

**Feature***	**ARA**	**sra-pipeline**	**pyrpipe**	**BICF SRA pipeline**	**magicblast** [7]	**bowtie2**
Data retrieval using SRA-toolkit	+	+	+	+	+	+
Data retrieval from cloud storage	+	-	-	-	-	-
Choice of the aligner	+	-	-	-	-	-

Additional sequence analysis tools	+	-	-	-	-	-
Screening mode using a fraction of a record	+	-	-	-	-	-
Adapter trimming	+	+	+	-	-	-
Data quality checks	+	-	-	+	-	-
Tabular metadata output	+	-	-	-	-	-
Alignment summary	+	-	-	-	-	-
Continuation of interrupted analysis	+	-	+	-	-	-

* *plus sign indicates implemented feature and minus sign marks missing feature;*

Output format

The results of each computational step are organized into distinct folders, optionally allowing easy manual exploration of the data. Each SRA accession's run- and sample-level attributes (e.g., run accession number, run type, total spots, study accession number, title, abstract, etc) are stored in dedicated files in the output directory. Upon completion of the analysis, the pipeline also generates a combined summary file sorted by the overall alignment percentage in decreasing order (**Supplementary Table 1**) along with the metadata for every SRA accession. This way of ranking the analyzed SRA records should help a user to select samples that most accurately reflect the sequences of interest for other downstream analyses. The summary file can also be regenerated by executing the analysis in the "summary" mode. The ARA tool is based on commonly used and tested programs, hence its performance solely depends on the hardware specifications and the quality of the network connection.

Examples of applications

Applying the 'screen' mode to a set of selected accessions is the simplest way to identify samples (regardless of their record annotation) that contain sequences of interest. Therefore, while simultaneously saving time and storage space, working with a fraction of the data in

‘screen’ mode enables the user to approximate whether the sequencing run contains any relevant reads. One of the most obvious examples of such usage is the identification of RNA-seq samples that contain transcripts corresponding to reference sequences. Such an analysis may provide data for expression experiments and/or supply information about tissues and/or conditions where the corresponding genes are expressed. The reference sequences may represent protein-coding or non-coding genes. The current version of the ARA pipeline also contains a dedicated module for fast screening of contamination in samples using Kraken2. It can be useful, for example, during an analysis of eukaryotic samples to detect sequences of bacterial or fungal origin. More tools will be added to the analysis step of the ARA pipeline in the future, enhancing its application range.

A case study: search for tRNA transcripts in Arabidopsis

To test run the ARA pipeline, we screened 100 selected RNA-seq SRA runs to identify samples that can be used to assess the expression of tRNA genes in *Arabidopsis thaliana*. For testing purposes, we downloaded the tRNA sequences for *A. thaliana* from the GtRNA database (TAIR10) [22] and performed the analyses using BLASTn and BOWTIE2. We have identified 57 samples that showed at least 1% content of tRNA-related transcripts (12 with >5% threshold) (**Supplementary Table 1**). As expected, most of the top-scoring samples represent results related to tRNA research. However, these are closely followed and intermixed with SRA records that contain high counts of tRNA-related reads and are not annotated as tRNA-containing samples. Minor differences between the BLASTn and BOWTIE2 mapping results refer mostly to sequences containing low-complexity fragments (top-scoring examples are shown in **Supplementary Figures 1 and 2**).

One of the major factors influencing the efficiency of the ARA pipeline ‘screen’ mode is the fraction of the record that will be downloaded and analyzed. Although this parameter depends on the type of used query sequences and the specific characteristics of the selected NGS records, we attempted to calculate the false negative rate using our tRNA example and expanded set of SRA records (205 samples). Our results show (Supplementary Table 2) that even at the lowest threshold (5%) only a small fraction (FNR = 0.073) did not pass the criterion of 1% tRNA sequence content. All the misclassified samples came from the same experiment (SRA ID SRP267192) and demonstrate the need for careful selection of the screening subset size depending on the project goals.

Conclusions

The ARA pipeline offers an easy and flexible way to explore data stored in SRA database records. It uses well-established tools in the NGS data processing and offers complete control over all the steps of the analysis. The pipeline provides a user-friendly and automated interface, starting from installation and ending with the creation of the combined summary file. Compared to other currently available tools (**Table 1**), the ARA tool incorporates a wider spectrum of possible analyses and options. The sample case of screening the RNA-seq records for tRNA transcripts demonstrates that the application of the ARA pipeline allows exploration of the SRA data beyond the information provided in the records' metadata.

Availability and Requirements

Project name: ARA pipeline

Project home page: https://github.com/maurya-anand/ARA

Operating system(s): Linux (64-bit) and macOS

Programming language: Perl (v5.3)

Other requirements: Docker or Mamba

License: GNU GPL v3.0

Any restrictions to use by non-academics: none

Additional Files

Supplementary Figure 1: *A bar plot showing a comparison of the total count of the reads mapped to tRNA reference by Bowtie2 and BLASTn for each tested SRA sample.*

Supplementary Figure 2: *A bar plot showing a comparison of the total execution time for Bowtie2 and BLASTn for each tested SRA sample.*

Supplementary Table 1: *An example of the summary table generated by the ARA pipeline showing selected columns related to alignment statistics and run information.*

Supplementary File 1: *Calculation of False Negative Rate values with BLASTn and BOWTIE2 using 5% incremental sample sizes.*

Data Availability

All data used in this study are available in the NCBI Sequence Read Archive (SRA) database.

Competing interests

The authors declare that they have no competing interests

Funding:

This work was supported by a grant from the National Science Center (NCN) 2017/27/B/NZ2/00467.

Authors' contributions

AM implemented the tool and performed calculations; MS supervised calculations and edited the manuscript; WMK designed the study, supervised the development of the tools and calculations, and wrote the manuscript. All authors have read and approved the final version of the manuscript.

Acknowledgments:

The computations were performed at the Poznan Supercomputing and Networking Center (PSNC) under grant numbers 312 and 528.

References:

1. Pearson WR. Rapid and sensitive sequence comparison with FASTP and FASTA. *Methods Enzymol*. 183:63–981990;

2. Altschul SF, Gish W, Miller W, Myers EW, Lipman DJ. Basic local alignment search tool. *J Mol Biol*. Elsevier BV; 215:403–101990;

3. Langmead B, Trapnell C, Pop M, Salzberg SL. Ultrafast and memory-efficient alignment of short DNA sequences to the human genome. *Genome Biol*. 10:R252009;

4. Li H, Durbin R. Fast and accurate short read alignment with Burrows-Wheeler transform. *Bioinformatics*. 25:1754–602009;

5. Katz K, Shutov O, Lapoint R, Kimelman M, Brister JR, O'Sullivan C. The Sequence Read Archive: a decade more of explosive growth. *Nucleic Acids Res*. 50:D387–902022;

6. Leinonen R, Akhtar R, Birney E, Bower L, Cerdeno-Tárraga A, Cheng Y, et al.. The European Nucleotide Archive. *Nucleic Acids Res*. 39:D28–312011;

7. Boratyn GM, Thierry-Mieg J, Thierry-Mieg D, Busby B, Madden TL. Magic-BLAST, an accurate RNA-seq aligner for long and short reads. *BMC Bioinformatics*. 20:4052019;

8. Langmead B, Salzberg SL. Fast gapped-read alignment with Bowtie 2. *Nat Methods*. 9:357–92012;

9. Singh U, Li J, Seetharam A, Wurtele ES. pyrpipe: a Python package for RNA-Seq workflows. *NAR Genom Bioinform*. Oxford Academic; 3:lqab0492021;

10. Wood DE, Lu J, Langmead B. Improved metagenomic analysis with Kraken 2. *Genome*

Biol. 20:2572019;

11. Kans J. Entrez Direct: E-utilities on the Unix Command Line. *Entrez Programming Utilities Help [Internet]*. National Center for Biotechnology Information (US);

12. Babraham Bioinformatics - FastQC A Quality Control tool for High Throughput Sequence Data. http://www.bioinformatics.babraham.ac.uk/projects/fastqc/ Accessed 2022 Oct 20.

13. Bolger AM, Lohse M, Usadel B. Trimmomatic: a flexible trimmer for Illumina sequence data. *Bioinformatics*. 30:2114–202014;

14. Mills WT 4th, Eadara S, Jaffe AE, Meffert MK. SCRAP: a bioinformatic pipeline for the analysis of small chimeric RNA-seq data. *RNA*. 2022; doi: 10.1261/rna.079240.122.

15. Czech L, Exposito-Alonso M. grenepipe: a flexible, scalable and reproducible pipeline to automate variant calling from sequence reads. *Bioinformatics*. 38:4809–112022;

16. Lopez-Delisle L, Rabbani L, Wolff J, Bhardwaj V, Backofen R, Grüning B, et al.. pyGenomeTracks: reproducible plots for multivariate genomic datasets. *Bioinformatics*. Oxford Academic; 37:422–32020;

17. Riehl K, Riccio C, Miska EA, Hemberg M. TransposonUltimate: software for transposon classification, annotation and detection. *Nucleic Acids Res*. 50:e642022;

18. Ye C, Thornlow B, Hinrichs A, Kramer A, Mirchandani C, Torvi D, et al.. matOptimize: A parallel tree optimization method enables online phylogenetics for SARS-CoV-2. *Bioinformatics*. 2022; doi: 10.1093/bioinformatics/btac401.

19. Goel M, Schneeberger K. plotsr: visualizing structural similarities and rearrangements between multiple genomes. *Bioinformatics*. 38:2922–62022;

20. Mitrofanov A, Ziemann M, Alkhnbashi OS, Hess WR, Backofen R. CRISPRtracrRNA: robust approach for CRISPR tracrRNA detection. *Bioinformatics*. 38:ii42–82022;

21. Wood DE, Lu J, Langmead B. Improved metagenomic analysis with Kraken 2. *Genome Biol*. 20:2572019;

22. Chan PP, Lowe TM. GtRNAdb 2.0: an expanded database of transfer RNA genes identified in complete and draft genomes. *Nucleic Acids Res*. 44:D184–92016;

Supplementary Figure 1

Click here to access/download
Supplementary Material
Supplementary Figure 1.pdf

Supplementary Figure 2

Click here to access/download
Supplementary Material
Supplementary Figure 2.pdf

Supplementary Table 1

Click here to access/download
Supplementary Material
Supplementary Table 1.xlsx

Supplementary File 1

Click here to access/download
Supplementary Material
Supplementary File 1.xlsx
