# Supplementary material for: ARA: a flexible pipeline for automated exploration of NCBI SRA datasets
Source: Gigascience. 2023 Aug 17;12:giad067. doi: 10.1093/gigascience/giad067 (PMC10433097; doi:10.1093/gigascience/giad067)
Supplement: giad067_GIGA-D-23-00049_Revision_2 [file giad067_giga-d-23-00049_revision_2.pdf]

GigaScience

ARA: A flexible pipeline for automated exploration of NCBI SRA datasets

--Manuscript Draft--

**Manuscript Number:**	GIGA-D-23-00049R2
**Full Title:**	ARA: A flexible pipeline for automated exploration of NCBI SRA datasets
**Article Type:**	Technical Note
**Funding Information:**	Narodowe Centrum Nauki (2017/27/B/NZ2/00467) — Prof. Wojciech Karlowski
**Abstract:**	Background: One of the most effective and useful methods to explore the content of biological databases is searching with nucleotide or protein sequences as a query. However, especially in the case of nucleic acids, due to the large volume of data generated by the Next Generation Sequencing technologies, this approach is often not available. The hierarchical organization of the NGS records is primarily designed for browsing or text-based searches of the information provided in metadata-related keywords, limiting the efficiency of database exploration.<br>Findings: We developed an automated pipeline that incorporates the well-established NGS data processing tools and procedures to allow easy and effective sampling of the NCBI SRA database records. Given a file with query nucleotide sequences, our tool estimates the matching content of SRA accessions by probing only a user-defined fraction of a record’s sequences. Based on the selected parameters, it allows performing a full mapping experiment with records that meet the required criteria. The pipeline is designed to be easy to operate — it offers a fully automatic setup procedure and is fixed on tested supporting tools. The modular design and implemented usage modes allow a user to scale up the analyses into complex computational infrastructure.<br>Conclusions: We present an easy-to-operate and automated tool that expands the way a user can access and explore the information contained within the records deposited in the NCBI SRA database.
**Corresponding Author:**	Wojciech Karlowski<br>Adam Mickiewicz University in Poznan<br>Poznan, POLAND
**Corresponding Author Secondary Information:**	
**Corresponding Author's Institution:**	Adam Mickiewicz University in Poznan
**Corresponding Author's Secondary Institution:**	
**First Author:**	Anand Maurya
**First Author Secondary Information:**	
**Order of Authors:**	Anand Maurya<br>Maciej Szymanski<br>Wojciech Karlowski
**Order of Authors Secondary Information:**	
**Response to Reviewers:**	Response to the Reviewer comment:<br><br>Reviewer #2:<br><br>C: In the newly added text before the conclusion, the authors mention that the FNR results are in Supplementary Table 2. However, as far as I understand, the results are in Supplementary File 1.<br><br>A: We are sorry for this silly error. It is now corrected in the new version of the manuscript.
**Additional Information:**	

Powered by Editorial Manager® and ProduXion Manager® from Aries Systems Corporation

Question	Response
Are you submitting this manuscript to a special series or article collection?	No
**Experimental design and statistics**<br><br>Full details of the experimental design and statistical methods used should be given in the Methods section, as detailed in our Minimum Standards Reporting Checklist. Information essential to interpreting the data presented should be made available in the figure legends.<br><br>Have you included all the information requested in your manuscript?	Yes
**Resources**<br><br>A description of all resources used, including antibodies, cell lines, animals and software tools, with enough information to allow them to be uniquely identified, should be included in the Methods section. Authors are strongly encouraged to cite Research Resource Identifiers (RRIDs) for antibodies, model organisms and tools, where possible.<br><br>Have you included the information requested as detailed in our Minimum Standards Reporting Checklist?	Yes
**Availability of data and materials**<br><br>All datasets and code on which the conclusions of the paper rely must be either included in your submission or deposited in publicly available repositories (where available and ethically appropriate), referencing such data using a unique identifier in the references and in the "Availability of Data and Materials" section of your manuscript.	Yes

Powered by Editorial Manager® and ProduXion Manager® from Aries Systems Corporation

Have you have met the above requirement as detailed in our Minimum Standards Reporting Checklist?	

Manuscript Click here to access/download;Manuscript;ARA manuscript - revision 2.docx

ARA: A flexible pipeline for automated exploration of NCBI SRA datasets

Anand Maurya

Department of Computational Biology, Institute of Molecular Biology and Biotechnology, Faculty of Biology, Adam Mickiewicz University in Poznan, Uniwersytetu Poznanskiego 6, 61-614 Poznan, Poland

email: anamau@amu.edu.pl

Maciej Szymanski

Department of Computational Biology, Institute of Molecular Biology and Biotechnology, Faculty of Biology, Adam Mickiewicz University in Poznan, Uniwersytetu Poznanskiego 6, 61-614 Poznan, Poland

email: mszyman@amu.edu.pl

Wojciech M Karlowski*

Department of Computational Biology, Institute of Molecular Biology and Biotechnology, Faculty of Biology, Adam Mickiewicz University in Poznan, Uniwersytetu Poznanskiego 6, 61-614 Poznan, Poland

email: wmk@amu.edu.pl

*) corresponding author

Abstract

Background

One of the most effective and useful methods to explore the content of biological databases is searching with nucleotide or protein sequences as a query. However, especially in the case of nucleic acids, due to the large volume of data generated by the Next Generation Sequencing technologies, this approach is often not available. The hierarchical organization of the NGS records is primarily designed for browsing or text-based searches of the information provided in metadata-related keywords, limiting the efficiency of database exploration.

Findings

We developed an automated pipeline that incorporates the well-established NGS data processing tools and procedures to allow easy and effective sampling of the NCBI SRA database records. Given a file with query nucleotide sequences, our tool estimates the matching content of SRA accessions by probing only a user-defined fraction of a record's sequences. Based on the selected parameters, it allows performing a full mapping experiment with records that meet the required criteria. The pipeline is designed to be easy to operate — it offers a fully automatic setup procedure and is fixed on tested supporting tools. The modular design and implemented usage modes allow a user to scale up the analyses into complex computational infrastructure.

Conclusions

We present an easy-to-operate and automated tool that expands the way a user can access and explore the information contained within the records deposited in the NCBI SRA database.

Keywords: SRA database, NGS data, database searching, sequence analysis

Background

The development of new computational tools dedicated to exploring the content of the protein and nucleotide sequence repositories revolutionized the methods of biological data access. FASTA [1] and BLAST [2] algorithms enable fast and efficient search of large databases for similar sequences and a statistical evaluation of the results. The heuristic algorithms used by these tools are much faster than implementations of rigorous dynamic programming algorithms. These methods, however, have a limited application in the context of large data

sets generated by high throughput Next Generation Sequencing (NGS) technologies. To meet the requirements of the NGS data processing, new approaches and specialized tools had to be developed.

The explosion of new techniques and algorithms for NGS sequence mapping led to the development of specialized solutions (e.g., BOWTIE [3] and BWA [4]) that can process sequence data more efficiently (in terms of required computational time) than the classical methods of sequence data exploration (e.g., BLAST and FASTA). However, although more effective, the new programs do not offer an easily accessible way for sequence-based searching of the NGS data repositories (e.g., Sequence Read Archive - SRA [5] or European Nucleotide Archive - ENA [6]). Due to the large volume of data and huge computational time required for sequence searches, exploration of the NGS databases is routinely restricted to text-based surveys with keywords and phrases provided in records' metadata. This is an obvious limitation since the user must depend on the completeness and correctness of the information provided in record descriptions.

The sequence-based screening of the whole SRA database records has been implemented recently in Magic-BLAST tool [7], designed specifically for mapping of large next-generation RNA or DNA sequencing runs. This functionality has been also incorporated, to some extent, into Bowtie2 since release 2.3.5 [8]. The availability of an option to automatically fetch SRA records is reported in prepackaged builds or alternatively, requires the source code compilation using the SRA software dependencies. A similar approach is offered by the *sra-pipeline* tool (https://github.com/FredHutch/sra-pipeline). Here, the program automates the process of downloading, mapping (BOWTIE2 [8]), compressing, and storing the results within the AWS cloud computing infrastructure. More advanced functionality, providing automated access to SRA records, is available in the *pyrpipe* Python package [9]. This tool helps, in a very convenient way, to incorporate the ability for automated RNA-seq data download into a custom pipeline. It also provides a programming interface for tools designed for basic sequence processing applications (e.g., adapter trimming). The *BICF SRA Pipeline* was created with a similar aim, which allows bulk download of data and quality assessment (https://doi.org/10.5281/zenodo.3739788). All of these currently available tools are designed to work by downloading the entire record data (FASTQ formatted files), which in most cases is very expensive in terms of computational resources and storage.

We have developed an integrated, feature-rich, universal, flexible, and ready-to-use solution that allows access to the data using both the SRA-tools and SRA-cloud sources. It provides a full or partial SRA record analysis mode and a choice of the sequence screening method

(BLAST [2] and BOWTIE2 [8]) and taxonomic profiling (Kraken2 [10]). The modular design of the pipeline allows easy further expansion of the sequence analysis toolbox. The implemented procedure also provides basic quality checks, including the removal of adapters and filtering of reads. Along with sequence data processing the pipeline extracts metadata information for each of the SRA records. A simple configuration schema allows full control of the procedure workflow, including the option to resume interrupted analyses and effortless integration into the distributed computational infrastructure.

ARA pipeline workflow

Implementation and tools

Similarly to *Entrez Direct* (command line utilities used to access in a text-based way NCBI databases) [11], the ARA (Automated SRA Records Analysis) tool is implemented in Perl and designed to be used from the shell prompt. It employs the NCBI SRA toolkit (https://github.com/ncbi/sra-tools) to download the raw data in FASTQ format from the SRA database. NCBI Entrez programming utilities provide access to the sample-level metadata along with the location of the raw data stored in the cloud. In case of a problem with *fastq-dump* (SRA toolkit) tool-mediated data downloads, the pipeline is designed to use NCBI e-utilities to find a link to the original SRA file (in the native SRA format) located either at AWS, GCP, or NCBI's storage servers. In such a case, the files are retrieved using the *wget* program and the NGS reads are extracted with *fastq-dump*. The *fastq-dump* supports downloading a fraction of reads from the beginning of a particular sequencing run accession. Using this tool speeds up downloading and analysis processes, which saves time and disk usage. A newer utility, *fasterq-dump*, from SRA toolkit, unfortunately, does not possess all the features offered by *fastq-dump* and is not currently implemented in the ARA pipeline. We will update our software once the developers of SRA toolkit implement the option of partial download in *fasterq-dump*.

Sequence quality evaluation

In order to ensure the highest quality data for processing, the downloaded sequences are subjected to a thorough three-step quality check procedure. FastQC [12] is executed on both raw and adapter-trimmed FASTQ files (**Figure 1** - first and second quality check). Trimmomatic [13] is used to filter low-quality sequences and/or remove adapters, which may profoundly influence the reliability of the alignment and other downstream analyses. The redundant reads are clustered using the Fastx toolkit (http://hannonlab.cshl.edu/fastx_toolkit/)

to reduce the database volume for sequence search and to speed up the analyses. The unique reads are mapped to the query sample using NCBI BLAST command line utility (https://www.ncbi.nlm.nih.gov/books/NBK279690/) and/or BOWTIE2 (**Figure 1**). In addition, the pipeline allows the taxonomic classification of reads using the 'Kraken2' program. By default, the tool uses a viral genomic reference dataset. However, it is possible to use any custom 'Kraken2' database by supplying the required data and modifying the configuration file.

Installation

The pipeline can be effortlessly set up using either Docker or Mamba package manager (https://mamba.readthedocs.io/en/latest/) which is a C++-based implementation of Conda (https://conda.io/). The Mamba implementation offers a faster interface and uses *libsolv* to effectively resolve the dependencies. The use of a package manager guarantees seamless installation of the required software tools along with their dependencies and has been successfully used in many recent bioinformatics projects (e.g., SCRAP [14], *grenepipe* [15], *pyGenomeTracks* [16], *TransposonUltimate* [17], *matOptimize* [18], *plotsr* [19], *CRISPRtracrRNA* [20]. The Perl modules are automatically downloaded and installed by the setup script through CPAN (https://metacpan.org/).

Results and discussion

Workflow modes

The ARA pipeline allows analyses in two major modes: 'screening' and 'full'. Both modes include the same steps (**Figure 1**) that involve basic procedures in NGS sequence data analysis. In the 'screening' mode the ARA tool will perform the analysis only on a small fraction (by default 5%) of sequences from each record. This step is useful in the selection of the best possible candidate datasets for further analyses. The 'full' mode allows downloading and processing of the entire sequence record. The 'both' analysis mode automates the two mentioned steps (first 'screening' and then 'full' analysis) (**Figure 1**). An additional 'summary' mode combines results of independently performed analyses (e.g., on computational grid structure) into one summary file that can be examined and used in subsequent steps (e.g., 'full' mode).

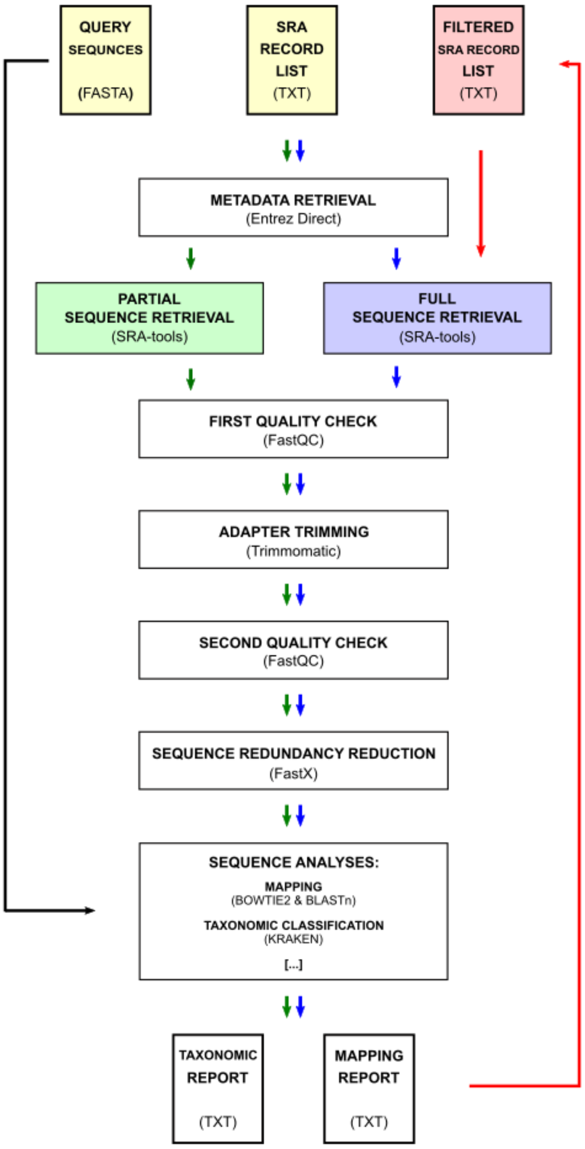

Figure 1. ***Graphic presentation of the ARA pipeline workflow***: *green arrows indicate steps run in the 'screen' mode. Blue arrows indicate steps executed in '`full' mode. Red arrows show the analysis path specific for the combined "both" mode (including automated generation of filtered SRA records list - depicted by a red rectangle). Yellow rectangles indicate user input data (query sequences and list of SRA records' accessions). White rectangles represent distinct steps in the analysis process (with tools indicated in brackets) and output reports generated by*

the pipeline. 'Sequence analyses' currently allow mapping and taxonomic classification, but can be easily expanded to include more tools (depicted by ellipsis in square brackets).

On each SRA 'Run ID' listed in the input file, the ARA pipeline performs data retrieval, adapter trimming, quality check, and mapping of reads using the reference sequences (**Figure 1**).

Features comparison

One of the most important features (**Table 1**) of the ARA pipeline is its modular architecture. Such a design allows an easy expansion of the available toolbox for downstream sequence analyses. In order to demonstrate this functionality, besides sequence mapping tools, we incorporated a module for the taxonomic classification of the reads using Kraken2 [21]. The pipeline generates a classification report for each sample using a provided reference database (viral genomes by default).

In case of problems with downloading the records, the pipeline attempts to retrieve the raw FASTQ file thrice before searching with NCBI e-utilities for alternate SRA file locations in the cloud storage. After successful retrieval of the SRA record, it extracts the single or paired-end reads using the *fastq-dump* utility (SRA toolkit). If both methods to download the data fail, the ARA pipeline logs the event and skips that particular SRA run accession. Following the data download step, the ARA tool performs quality checks to ensure that the absence of matching sequences is not related to sequencing errors. The parameters of each of the analysis steps can be tweaked through the ARA configuration file. A user is required to provide a list of SRA run accessions as the starting parameter for the analysis.

Table 1. *Comparison of the ARA pipeline features with other similar tools.*

**Feature***	**ARA**	**sra-pipeline**	**pyrpipe**	**BICF SRA pipeline**	**magicblast** [7]	**bowtie2**
Data retrieval using SRA-toolkit	+	+	+	+	+	+
Data retrieval from cloud storage	+	-	-	-	-	-
Choice of the aligner	+	-	-	-	-	-

Additional sequence analysis tools	+	-	-	-	-	-
Screening mode using a fraction of a record	+	-	-	-	-	-
Adapter trimming	+	+	+	-	-	-
Data quality checks	+	-	-	+	-	-
Tabular metadata output	+	-	-	-	-	-
Alignment summary	+	-	-	-	-	-
Continuation of interrupted analysis	+	-	+	-	-	-

* *plus sign indicates implemented feature and minus sign marks missing feature;*

Output format

The results of each computational step are organized into distinct folders, optionally allowing easy manual exploration of the data. Each SRA accession's run- and sample-level attributes (e.g., run accession number, run type, total spots, study accession number, title, abstract, etc) are stored in dedicated files in the output directory. Upon completion of the analysis, the pipeline also generates a combined summary file sorted by the overall alignment percentage in decreasing order (**Supplementary Table 1**) along with the metadata for every SRA accession. This way of ranking the analyzed SRA records should help a user to select samples that most accurately reflect the sequences of interest for other downstream analyses. The summary file can also be regenerated by executing the analysis in the "summary" mode. The ARA tool is based on commonly used and tested programs, hence its performance solely depends on the hardware specifications and the quality of the network connection.

Examples of applications

Applying the 'screen' mode to a set of selected accessions is the simplest way to identify samples (regardless of their record annotation) that contain sequences of interest. Therefore, while simultaneously saving time and storage space, working with a fraction of the data in

‘screen’ mode enables the user to approximate whether the sequencing run contains any relevant reads. One of the most obvious examples of such usage is the identification of RNA-seq samples that contain transcripts corresponding to reference sequences. Such an analysis may provide data for expression experiments and/or supply information about tissues and/or conditions where the corresponding genes are expressed. The reference sequences may represent protein-coding or non-coding genes. The current version of the ARA pipeline also contains a dedicated module for fast screening of contamination in samples using Kraken2. It can be useful, for example, during an analysis of eukaryotic samples to detect sequences of bacterial or fungal origin. More tools will be added to the analysis step of the ARA pipeline in the future, enhancing its application range.

A case study: search for tRNA transcripts in Arabidopsis

To test run the ARA pipeline, we screened 100 selected RNA-seq SRA runs to identify samples that can be used to assess the expression of tRNA genes in *Arabidopsis thaliana*. For testing purposes, we downloaded the tRNA sequences for *A. thaliana* from the GtRNA database (TAIR10) [22] and performed the analyses using BLASTn and BOWTIE2. We have identified 57 samples that showed at least 1% content of tRNA-related transcripts (12 with >5% threshold) (**Supplementary Table 1**). As expected, most of the top-scoring samples represent results related to tRNA research. However, these are closely followed and intermixed with SRA records that contain high counts of tRNA-related reads and are not annotated as tRNA-containing samples. Minor differences between the BLASTn and BOWTIE2 mapping results refer mostly to sequences containing low-complexity fragments (top-scoring examples are shown in **Supplementary Figures 1 and 2**).

One of the major factors influencing the efficiency of the ARA pipeline ‘screen’ mode is the fraction of the record that will be downloaded and analyzed. Although this parameter depends on the type of used query sequences and the specific characteristics of the selected NGS records, we attempted to calculate the false negative rate using our tRNA example and expanded set of SRA records (205 samples). Our results show (Supplementary File 1) that even at the lowest threshold (5%) only a small fraction (FNR = 0.073) did not pass the criterion of 1% tRNA sequence content. All the misclassified samples came from the same experiment (SRA ID SRP267192) and demonstrate the need for careful selection of the screening subset size depending on the project goals.

Conclusions

The ARA pipeline offers an easy and flexible way to explore data stored in SRA database records. It uses well-established tools in the NGS data processing and offers complete control over all the steps of the analysis. The pipeline provides a user-friendly and automated interface, starting from installation and ending with the creation of the combined summary file. Compared to other currently available tools (**Table 1**), the ARA tool incorporates a wider spectrum of possible analyses and options. The sample case of screening the RNA-seq records for tRNA transcripts demonstrates that the application of the ARA pipeline allows exploration of the SRA data beyond the information provided in the records' metadata.

Availability and Requirements

Project name: ARA pipeline

Project home page: https://github.com/maurya-anand/ARA

Operating system(s): Linux (64-bit) and macOS

Programming language: Perl (v5.3)

Other requirements: Docker or Mamba

License: GNU GPL v3.0

Any restrictions to use by non-academics: none

Additional Files

Supplementary Figure 1: *A bar plot showing a comparison of the total count of the reads mapped to tRNA reference by Bowtie2 and BLASTn for each tested SRA sample.*

Supplementary Figure 2: *A bar plot showing a comparison of the total execution time for Bowtie2 and BLASTn for each tested SRA sample.*

Supplementary Table 1: *An example of the summary table generated by the ARA pipeline showing selected columns related to alignment statistics and run information.*

Supplementary File 1: *Calculation of False Negative Rate values with BLASTn and BOWTIE2 using 5% incremental sample sizes.*

Data Availability

All data used in this study are available in the NCBI Sequence Read Archive (SRA) database.

Competing interests

The authors declare that they have no competing interests

Funding:

This work was supported by a grant from the National Science Center (NCN) 2017/27/B/NZ2/00467.

Authors' contributions

AM implemented the tool and performed calculations; MS supervised calculations and edited the manuscript; WMK designed the study, supervised the development of the tools and calculations, and wrote the manuscript. All authors have read and approved the final version of the manuscript.

Acknowledgments:

The computations were performed at the Poznan Supercomputing and Networking Center (PSNC) under grant numbers 312 and 528.

References:

1. Pearson WR. Rapid and sensitive sequence comparison with FASTP and FASTA. *Methods Enzymol*. 183:63–981990;

2. Altschul SF, Gish W, Miller W, Myers EW, Lipman DJ. Basic local alignment search tool. *J Mol Biol*. Elsevier BV; 215:403–101990;

3. Langmead B, Trapnell C, Pop M, Salzberg SL. Ultrafast and memory-efficient alignment of short DNA sequences to the human genome. *Genome Biol*. 10:R252009;

4. Li H, Durbin R. Fast and accurate short read alignment with Burrows-Wheeler transform. *Bioinformatics*. 25:1754–602009;

5. Katz K, Shutov O, Lapoint R, Kimelman M, Brister JR, O'Sullivan C. The Sequence Read Archive: a decade more of explosive growth. *Nucleic Acids Res*. 50:D387–902022;

6. Leinonen R, Akhtar R, Birney E, Bower L, Cerdeno-Tárraga A, Cheng Y, et al.. The European Nucleotide Archive. *Nucleic Acids Res*. 39:D28–312011;

7. Boratyn GM, Thierry-Mieg J, Thierry-Mieg D, Busby B, Madden TL. Magic-BLAST, an accurate RNA-seq aligner for long and short reads. *BMC Bioinformatics*. 20:4052019;

8. Langmead B, Salzberg SL. Fast gapped-read alignment with Bowtie 2. *Nat Methods*. 9:357–92012;

9. Singh U, Li J, Seetharam A, Wurtele ES. pyrpipe: a Python package for RNA-Seq workflows. *NAR Genom Bioinform*. Oxford Academic; 3:lqab0492021;

10. Wood DE, Lu J, Langmead B. Improved metagenomic analysis with Kraken 2. *Genome*

Biol. 20:2572019;

11. Kans J. Entrez Direct: E-utilities on the Unix Command Line. *Entrez Programming Utilities Help [Internet]*. National Center for Biotechnology Information (US);

12. Babraham Bioinformatics - FastQC A Quality Control tool for High Throughput Sequence Data. http://www.bioinformatics.babraham.ac.uk/projects/fastqc/ Accessed 2022 Oct 20.

13. Bolger AM, Lohse M, Usadel B. Trimmomatic: a flexible trimmer for Illumina sequence data. *Bioinformatics*. 30:2114–202014;

14. Mills WT 4th, Eadara S, Jaffe AE, Meffert MK. SCRAP: a bioinformatic pipeline for the analysis of small chimeric RNA-seq data. *RNA*. 2022; doi: 10.1261/rna.079240.122.

15. Czech L, Exposito-Alonso M. grenepipe: a flexible, scalable and reproducible pipeline to automate variant calling from sequence reads. *Bioinformatics*. 38:4809–112022;

16. Lopez-Delisle L, Rabbani L, Wolff J, Bhardwaj V, Backofen R, Grüning B, et al.. pyGenomeTracks: reproducible plots for multivariate genomic datasets. *Bioinformatics*. Oxford Academic; 37:422–32020;

17. Riehl K, Riccio C, Miska EA, Hemberg M. TransposonUltimate: software for transposon classification, annotation and detection. *Nucleic Acids Res*. 50:e642022;

18. Ye C, Thornlow B, Hinrichs A, Kramer A, Mirchandani C, Torvi D, et al.. matOptimize: A parallel tree optimization method enables online phylogenetics for SARS-CoV-2. *Bioinformatics*. 2022; doi: 10.1093/bioinformatics/btac401.

19. Goel M, Schneeberger K. plotsr: visualizing structural similarities and rearrangements between multiple genomes. *Bioinformatics*. 38:2922–62022;

20. Mitrofanov A, Ziemann M, Alkhnbashi OS, Hess WR, Backofen R. CRISPRtracrRNA: robust approach for CRISPR tracrRNA detection. *Bioinformatics*. 38:ii42–82022;

21. Wood DE, Lu J, Langmead B. Improved metagenomic analysis with Kraken 2. *Genome Biol*. 20:2572019;

22. Chan PP, Lowe TM. GtRNAdb 2.0: an expanded database of transfer RNA genes identified in complete and draft genomes. *Nucleic Acids Res*. 44:D184–92016;

Supplementary Figure 1

Click here to access/download
Supplementary Material
Supplementary Figure 1.pdf

Supplementary Figure 2

Click here to access/download
Supplementary Material
Supplementary Figure 2.pdf

Supplementary Table 1

Click here to access/download
Supplementary Material
Supplementary Table 1.xlsx

Supplementary File 1

Click here to access/download
Supplementary Material
Supplementary File 1.xlsx
