# Supplementary figures and images for: ARA: a flexible pipeline for automated exploration of NCBI SRA datasets
Source: Gigascience. 2023 Aug 17;12:giad067. doi: 10.1093/gigascience/giad067 (PMC10433097; doi:10.1093/gigascience/giad067)

Total hits

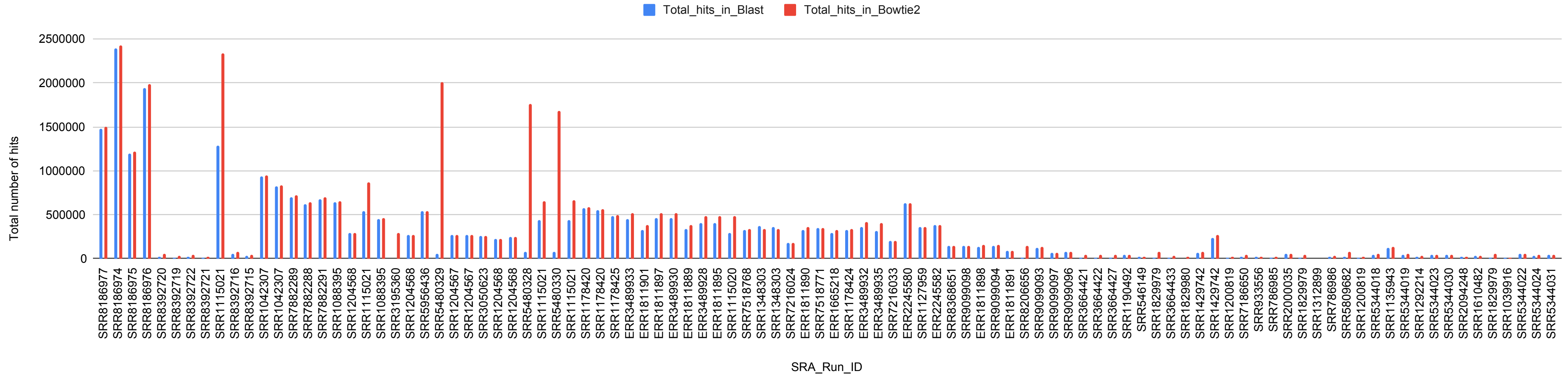

Supplement: giad067_Supplemental_Figures_and_Tables [file giad067_supplemental_figures_and_tables.zip › Supplementary Figure 1.pdf]

Execution time (seconds)

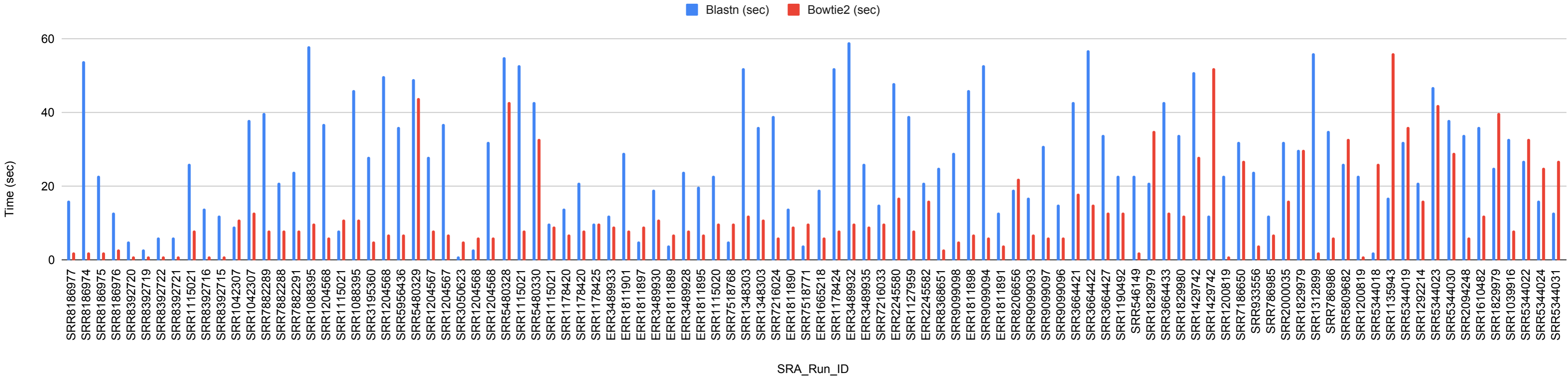

Supplement: giad067_Supplemental_Figures_and_Tables [file giad067_supplemental_figures_and_tables.zip › Supplementary Figure 2.pdf]
