# Supplementary material for: ARA: a flexible pipeline for automated exploration of NCBI SRA datasets
Source: Gigascience. 2023 Aug 17;12:giad067. doi: 10.1093/gigascience/giad067 (PMC10433097; doi:10.1093/gigascience/giad067)
Supplement: giad067_Response_to_Reviewer_Comments_Original_Submission [file giad067_response_to_reviewer_comments_original_submission.pdf]

Author's Response To Reviewer Comments

Close

Dear Prof. Nogoy,

Thank you very much for considering our article entitled “ARA: A flexible pipeline for automated exploration of NCBI SRA datasets” for publication in GigaScience. Your opinion and the comments of the Reviewers have stimulated a number of changes to the manuscript that we think have further improved the article.

In the revised version of the manuscript and the responses, we have addressed all the issues raised by the Reviewers. In summary, we have investigated the influence of the screening sample size on the false discovery rate of the predictions and introduced several modifications to the documentation and manuscript providing more information related to the used tools and computational workflow.

All changes to the manuscript are indicated in the text with red color.

Below are our point-by-point responses (A) to all the reviewers’ comments (C).

Sincerely,

Wojciech Karlowski

Responses to Reviewers’ comments:

Reviewer #1: This package runs a pipeline that handles many of the tasks that a user might want to perform on SRA sequences, including data retrieval, data quality checks, clustering redundant reads, etc. I think the most interesting feature is the ability to search a subset of an SRA run in order to judge it's relevance to a particular problem.

We would like to thank the Reviewer for carefully reading our manuscript and providing comments that address problems related to the presented work.

C: I have some comments/requests.

I think the authors need to provide more details about the screening feature:

In screening mode, 10% of the sequences from a record are downloaded and aligned (by default). I assume this is the first 10% of the reads, as opposed to every 10th read. The authors should state this explicitly. Also, a comment on whether or not looking at only the first 10% might be a non-representative sample would be good.

A: The reads sampling functionality is provided by the fastq-dump program (SRA-toolkit) and according to documentation it fetches the first 10% of the reads. We now provide this information in the manuscript text.

C: I'm confused about what's being shown in suppl. figures 1 and 2. I think these are alignments for all the sequences in the record, though I'm not 100% sure. I think the authors would want to show a comparison of the prediction that results from sampling 10% of the reads and the full run. That is, can they demonstrate, even anecdotally, that this feature works and is valuable? This is important for readers to take the article seriously.

A: These two figures are provided in the manuscript to illustrate the efficiency (sensitivity and computational effectiveness) of two implemented in the ARA pipeline sequence comparison tools (Bowtie2 and BLASTn). Our intention was to present significant differences in the output results related to the used tool (expected) and tested sample (unexpected). We have slightly modified the caption of these figures to state our intentions more clearly.
The current version of the manuscript is also supplemented with the FDR analysis of the case study data set. In a new supplementary data file, we provide detailed information about the efficiency of the sequence comparison tools in a spectrum of screening sampling thresholds, including the full dataset.

C: What is the tRNA database that is used to screen the records for the two suppl figures? It seems that should be available to readers and listed in the Data Availability section.

A: The tRNA sequences are sourced from the official annotation of the Arabidopsis genome located in GtRNAdb database (Arabidopsis thaliana - TAIR10). We have added information to the manuscript stating the source of the reference sequences in the application example.

C: How were the 100 SRA runs for the suppl. figures selected? Some were submitted by the last author (which I have no issue with), but I think it's important that the readers know the basis for the selection. Were they part of another project in the lab, were they chosen based on some other criteria that made them interesting, etc.?

A: The ARA pipeline is currently employed in a number of high-throughput screening projects in our lab. One of them deals with the expression of tRNA genes in Arabidopsis thaliana and consists of experimental as well as computational analyses. Hence, some of the samples that appeared on our test screening results are authored by us. This actual finding we consider as a positive control - we have expected that these samples should appear in the results. The original experiment involved screening more than 20 000 SRA accessions. For the presentation of differences between Bowtie2 and BLASTn screening, we have selected from these results representative examples to show the effect on the fragment identification efficiency and computational cost. Hence, these results should not be considered as a benchmark, but rather as a list of representative examples.

C: There are issues with the references. References 2 and 9 are the same. 7 and 10 are also the same, etc.

A: We are sorry for these errors. We corrected and manually verified the references in the revised version of the document.

Reviewer #2: The ARA tool is a very interesting gate for the preliminary exploration of a target sequence to the massive SRA repository. Using ARA, the user is able to more efficiently mine samples of relevance from SRA, as well as have initial insights on the spread of your target sequence. In addition, the tool's modularity should increase its usability. I particularly like the "both" exploration method. For these reasons, I recommend the publication of the ARA pipeline with some reviews. Congratulations on your work.

We would like to thank the Reviewer for positive feedback on our work.

Major Reviews:

C: The tool is an easy-to-use approach for preliminary sequence exploration. However, it is not novel. Although its user-friendliness is a selling point, stricter validation should be provided for the screening process.

A: We do agree that the concept of preliminary sequence screening is not novel. However, to our best knowledge, it was never implemented in the case of huge NGS sequence resources. Additionally, our

approach is very flexible - it allows switching sequence comparison programs (currently two implemented) and an easy expansion of the available toolbox by almost any additional tool. We provide in the introduction information about other tools that employ similar strategies and compare their functionalities in Table 1.

C: The screening approach downloads, by default, 10% of the sequence file from SRA to use with the query sequence. Although the authors allow the user to change the default value, no value is given as a recommendation, and no explanation is provided as to why 10% was chosen as the default cut-off.

A: We do agree that it is very difficult to establish one fixed parameter representing effectively the content of any sequence record. The suggested 10% threshold was mainly based on our practical observation - the value that was most useful in a wide spectrum of records and applications. Additionally, some of the recent studies (e.g., https://doi.org/10.1186/s12859-022-04572-7 and https://doi.org/10.1016/j.xhgg.2022.100103) demonstrate that 10% is a representative fraction of an NGS sequencing record.
However, based on the suggested by the Reviewer FDR analysis, in the updated version of the pipeline, we set as default the 5% value. For more explanation please see our answer below.

C: What exploratory bias would a user have if they only used the screening exploration method? What is the False Negative rate of the screening method, i.e., how often I won't find my sequence in an SRA sample simply by chance?

A: This is a very hard question to answer because the value will depend on the query sequence set, the inclusion criteria (positive or negative classification of a sample based on the percent of matching fragments), and the selection of the samples. However, following the Reviewer’s advice, we have performed the calculations on our test data set (207 SRA samples tested with tRNA transcripts from Arabidopsis thaliana) to gain a preliminary glimpse into the usefulness of the screening procedure. The results are now provided as a new Supplementary File 1 and show that the 5% threshold (lowest) provides good estimates at the FDR of 0.0727. Only 4 NGS runs (from experiment SRP267192) required a higher screening threshold value and behaved erratically during the analysis (for details please see Supplementary File 1).

C: I would like to see a recommendation for the cut-off percentage based on an optimal balance of False Negative and Storage efficiency exploration.

A: As stated in the previous answer, it may be impossible to assess a universal cut-off value that will represent all possible research scenarios (reference sequences and SRA records’ characteristics). Nevertheless, with further exploration of our tRNA example, we show the effect of the screening threshold value and provide the results in Supplementary File 1. From these analyses, we conclude that the lowest tested value (5%) is sufficient in most cases to successfully select the sample. However, even in this example, we have detected erratically behaving samples (SRR12008191-4). Therefore, the proposed by us default value should be considered merely as a suggestion and should be treated as a starting point for further exploration by a pipeline user.

C: In addition, how is your sampling done? Do you randomly download 10% of the sequences, or is there some sort of sampling pattern?

A: The sequence files hosted on NCBI SRA are submitted by the researchers. Based on their original order in the supplied file, we download the data using ‘-X’ option in fastq-dump tool. It's vital to note that the submitter may have sorted, aligned, transformed, or generated the fastq file randomly before uploading. To truly randomize the record content we would need to download the whole dataset, which is in obvious contrast with the idea of fast screening of the records.

C: In line 157, the authors mention downloading a "representative" fraction of the sequences from each record. However, there is no representative analysis shown.

A: We agree that this statement was misleading. We have corrected the sentence by removing the ‘representative’ adjective.

Minor Reviews:

C: Line 74 (… development of specialized solutions (e.g., BOWTIE [3] and BWA [4]) that can process sequence data more efficiently than the classical methods):

It is not clear what are the classical methods. Are you referring to FASTA and BLAST? Please make it more transparent. In addition, please provide a reference or metric that indicates the comparison of efficiency among the methods.

A: We are referring here to classical database exploration tools like BLAST and FASTA (introduced in the previous paragraph). We have modified the sentence to make it clear. We also explain in this sentence the mentioned efficiency metrics as the required computational time.

C: Line 80 (… This is an obvious limitation since the user must depend on the completeness and correctness of the information provided in record descriptions.):

Can you provide proof or indicate a reference that shows that depending on the records metadata is, in fact, a limitation?

A: There are instances where the information about the run in the SRA repository, as well as the matching sample-level attributes in the BioSample repository, is missing or incomplete. For example, the SRA accessions SRR499990, ERR2596563, and ERR1769645. If the metadata is inconsistent or incomplete, finding samples that contain sequences of interest might be difficult in NCBI SRA.

C: Line 119 (…In such a case, the files are retrieved using the wget program, and the NGS reads are extracted with fastq-dump. However, the fastq-dump supports downloading of a fraction of reads from a particular sequencing run accession.):

I don't understand the use of adversity in the sentence, i.e., 'However.' If I understand correctly, the ability to download only partial sequence is by design.

A: The adversity was placed here to contrast the functionality of fastq-dump and wget downloading modes. Following the Reviewer’s suggestion, we have removed it from the sentence.

C: Line 138 (… . By default, the tool uses a viral genomic reference dataset.):

Is there a particular reason why you choose this as default? Please provide a sentence justifying the selection of this default.

A: We are using the viral genomic database (release: 9/8/2022) because it's smaller in size and quicker to set up during the installation of the pipeline. A user can download a larger reference and use it in the pipeline after the installation is finished.

C: Line 142 (The whole Installation description)

Please provide the versions of all used tools as supplementary material. I understand that most of the tool's version can be found in the requirements file for the conda installation. However, it is relevant to reproducibility to provide the version of all used tools and dependencies clearly.

The manuscript installation section also does not clearly state the amount of computer resources needed to install ARA. Please provide the storage requirements for the tool itself as well as an estimation of how much extra storage is required after ARA generates the results.

A: Since the version of the used tools and required space for installation may change frequently, we provide now this information in the pipeline documentation.

C: Line 170, figure 1 legend (… Blue arrows indicate steps executed in "full' mode. Red arrows show the analysis path specific for the combined "screen + full" mode)

Instead of "screen + full" please use "both" as this was the name of the method previously introduced on line 160.

A: Thank you for spotting this oversight. We have corrected the figure caption accordingly.

C: Line 198, Table 1:

Please include in the table which of the tools is able to do partial data download. Or do you imply this in the "data screening mode"?

A: The ability to download partial data is referred to as “data screening mode”. The table has been modified to improve clarity.

C: The magicblast tool is not compared with the ARA tool in the introduction. Please do so.

A: We now include a short description of MagicBlast in the introduction.

C: Line 206 (… Upon completion of the analysis, the pipeline also generates a combined summary file sorted by the overall alignment percentage in decreasing order (Supplementary Table 1))

Please indicate, somehow, on the final results file which method ARA used at the end and that the rows are sorted.

A: The final table is sorted in decreasing order of the total percentage of alignment. It measures how many reads out of the total in the sample have been aligned by the aligner (BLASTn/BOWTIE2). Additionally, we now indicate the tool as well as the sorting status in the name of the output file.

C: Line 229 (A case study…):

Please include a comparison to the currently used approaches. How much more efficient is your screen mode? What would be the result of your exploration using the classical methods?

A: Unfortunately, none of the currently available methods that can process automatically SRA records does provide a ‘screening’ mode (please see Table 1). However, since all the other tools download the full record data and the mapping algorithms are common between them, we may assume that, on the same computational architecture, their time performance would be worse.

C: Line 255 (Programming language: Perl):

Please provide the version.

A: We provide now this information in the ‘Availability and Requirements’ section.

GitHub page and Installation procedure:

Installation:

C: Please provide detailed instructions on downloading and extracting the zip file. Which zip file? If I

understand, the more straightforward approach would be to use 'git clone' or download the complete repository as a zip using 'wget'.

When using git clone, the instructions should change to cd ARA-main
"'original
cd ARA/
mamba env create --file requirements.yaml
mamba activate ara_env
perl setup.pl
‘ ‘ ‘
"'edited
cd ARA-main/
mamba env create --file requirements.yaml
mamba activate ara_env
perl setup.pl
‘ ‘ ‘
A: Thank you for spotting this oversight. We have updated the instructions.

Errors and comments

C: #1
perl ara.pl --help
perl: warning: Setting locale failed.
perl: warning: Please check that your locale settings:
LANGUAGE = (unset),
LC_ALL = (unset),
LC_CTYPE = "UTF-8",
LANG = "en_US.UTF-8"
are supported and installed on your system.
perl: warning: Falling back to a fallback locale ("en_US.UTF-8").
Can't locate Config/Simple.pm in @INC (you may need to install the Config::Simple module) (@INC contains: /mnt/tools/miniconda3/envs/ara_env/lib/perl5/5.32/site_perl
/mnt/tools/miniconda3/envs/ara_env/lib/perl5/site_perl
/mnt/tools/miniconda3/envs/ara_env/lib/perl5/5.32/vendor_perl
/mnt/tools/miniconda3/envs/ara_env/lib/perl5/vendor_perl
/mnt/tools/miniconda3/envs/ara_env/lib/perl5/5.32/core_perl
/mnt/tools/miniconda3/envs/ara_env/lib/perl5/core_perl .) at ara.pl line 11.
BEGIN failed--compilation aborted at ara.pl line 11.

#1 Solution
I managed to solve this error by correcting my paths. I suggest you include in the troubleshooting section how to configure your Perl paths:

~/.cpan/CPAN/MyConfig.pm

Specifically, I had problems with my path from 'tar' and 'make.'

A: Thank you for this suggestion. We have created a troubleshooting section to address this and all other issues with the installation, configuration, and execution of the ARA tool.

C: #2

Can't locate Parallel/ForkManager.pm in @INC (you may need to install the Parallel::ForkManager module) (@INC contains: /mnt/tools/miniconda3/envs/ara_env/lib/perl5/5.32/site_perl

/mnt/tools/miniconda3/envs/ara_env/lib/perl5/site_perl
/mnt/tools/miniconda3/envs/ara_env/lib/perl5/5.32/vendor_perl
/mnt/tools/miniconda3/envs/ara_env/lib/perl5/vendor_perl
/mnt/tools/miniconda3/envs/ara_env/lib/perl5/5.32/core_perl
/mnt/tools/miniconda3/envs/ara_env/lib/perl5/core_perl .) at ara.pl line 12.
BEGIN failed--compilation aborted at ara.pl line 12.

#2 Solution
I could not fix this using only Perl. I fix it by installing the missing dependencies using conda/mamba:

mamba install -c bioconda perl-parallel-forkmanager

A: We have updated the troubleshooting section to include the solution to this configuration problem.

C: #3
If the warning messages are irrelevant, I would suppress them, as the log files become polluted.

A: We have added an option to suppress warning messages in the log.

C: #4

I would like a more detailed description of your output files—for instance, their naming conventions.

A: We have updated documentation to provide a description of the naming conventions used by the ARA tool.

C: #5
If some of your output files are results from other tools, please link the user to the file description from the original tool.

A: We have updated documentation to provide links to the tools implemented in the ARA pipeline.

C: #6
Indicate the storage results. From the example run, a 4MB file generated 592 MB results. An increase of 148 times. I understand that it depends on how many SRA samples the users want to explore, but it would be good to see some sort of estimation of required resources per average size of the reference sample.

A: We provide in the pipeline documentation additional information about storage statistics related to the presented example. This information may offer a user, not familiar with NGS data processing, a preliminary view into the storage needs of a particular project. Unfortunately, we are unable to provide any universal estimation of these values.

C: #7
From the Example run, sample SRR12548227 did not output all folders. Is this expected? Why?

My results were:

screening_results/SRR12548227:
fastQC raw_fastq trimmed_data

screening_results/SRR7289585:
blastn bowtie2 fastQC kraken2 raw_fastq trimmed_data

screening_results/SRR8392720:
blastn bowtie2 fastQC kraken2 raw_fastq trimmed_data

A: We have tried several times to reproduce this error without success. It might be related to the specific configuration of the operating system. We recommend checking the ‘runlog.SRR12548227.txt’ file generated in the output folder for any error messages. We provide this debugging hint in the troubleshooting section of the documentation.

C: #8
conf.txt file
For tool-specific parameters, please indicate where the user can find information about the parameter

A: We now provide this information in the pipeline documentation.

C: #9
I would like to have ara.pl on the PATH of my ara_env conda environment.
Instruct the user to add ara.pl to the conda PATH

A: We have added this information to the pipeline documentation.

C: #10

I got a relevant error while trying to install using docker. Please, check if the installation is correct.
Following is my error:

Err:16 http://archive.ubuntu.com/ubuntu jammy-updates/restricted amd64 Packages
#0 1038.3 Connection timed out [IP: 185.125.190.39 80]
#0 1038.4 Ign:17 http://archive.ubuntu.com/ubuntu jammy-updates/main amd64 Packages
#0 1038.4 Fetched 1185 kB in 17min 18s (1141 B/s)
#0 1038.4 Reading package lists...
#0 1038.4 E: Failed to fetch http://security.ubuntu.com/ubuntu/dists/jammy-security/restricted/binary-amd64/Packages Connection timed out [IP: 185.125.190.39 80]
#0 1038.4 E: Failed to fetch http://archive.ubuntu.com/ubuntu/dists/jammy/universe/binary-amd64/Packages Connection timed out [IP: 91.189.91.39 80]
#0 1038.4 E: Failed to fetch http://archive.ubuntu.com/ubuntu/dists/jammy-updates/restricted/binary-amd64/Packages Connection timed out [IP: 185.125.190.39 80]

A: We have checked the Docker image. The above-shown error messages indicate problems with the network connection or server availability. In such a case the installation procedure should be repeated. We have provided a short description of this error in the troubleshooting section of the documentation.

C: #11
Add an outputs section on the GitHub page explaining the expected outputs folder structure and files

A: We provide the description of the directory structure and file naming convention in the documentation (please see also answer to comment #4).

Close
