# Supplementary material for: ARA: a flexible pipeline for automated exploration of NCBI SRA datasets
Source: Gigascience. 2023 Aug 17;12:giad067. doi: 10.1093/gigascience/giad067 (PMC10433097; doi:10.1093/gigascience/giad067)
Supplement: giad067_Response_to_Reviewer_Comments_Revision_1 [file giad067_response_to_reviewer_comments_revision_1.pdf]

Author's Response To Reviewer Comments

Close

Response to the Reviewer comment:

Reviewer #2:

C: In the newly added text before the conclusion, the authors mention that the FNR results are in Supplementary Table 2. However, as far as I understand, the results are in Supplementary File 1.

A: We are sorry for this silly error. It is now corrected in the new version of the manuscript.

Close
