# Supplementary material for: ARA: a flexible pipeline for automated exploration of NCBI SRA datasets
Source: Gigascience. 2023 Aug 17;12:giad067. doi: 10.1093/gigascience/giad067 (PMC10433097; doi:10.1093/gigascience/giad067)
Supplement: giad067_Response_to_Reviewer_Comments_Revision_2 [file giad067_response_to_reviewer_comments_revision_2.pdf]

Author's Response To Reviewer Comments

Close

Dear Dr. Nogoy,

Thank you for reviewing our manuscript. We sincerely appreciate your valuable feedback and have incorporated the suggested changes:

- We have moved all URLs to the Bibliography section.
- We have supplemented the URLs with the Access Date to ensure accurate referencing.
- The ARA has been registered in the SciCrunch database, and we have included the RRID Number in the manuscript.
- We have also provided information about the location of accession numbers of SRA records used in the study. Due to their large number, we have not listed them directly in the manuscript.
- Furthermore, we have added a reference to ARA's GigaDB record.
- Additionally, we have registered ARA in the BioTools database and included the corresponding ID in the manuscript.

For easy reference all changes are marked in red color.

However, we encountered difficulties with the registration of ARA in the Workflowhub. The Workflowhub's main purpose is to store records that reference workflows operating in specific, dedicated environments. Our tool is a stand-alone program and does not operate within a workflow environment. Nevertheless, we attempted to register the tool, but the procedure requires manual verification of the development team, and unfortunately, we have not received any response so far. We will continue our efforts to get it registered if you believe that the Workflowhub entry is crucial for the publication of our manuscript.

Once again, we express our gratitude for your support and valuable input throughout this process.

Best regards,

Wojciech Karlowski

Close
