# Supplementary material for: ARA: a flexible pipeline for automated exploration of NCBI SRA datasets
Source: Gigascience. 2023 Aug 17;12:giad067. doi: 10.1093/gigascience/giad067 (PMC10433097; doi:10.1093/gigascience/giad067)
Supplement: giad067_Reviewer_1_Report_Original_Submission — Tom Madden -- 4/3/2023 Reviewed [file giad067_reviewer_1_report_original_submission.pdf]

Reviewer Report

Title: ARA: A flexible pipeline for automated exploration of NCBI SRA datasets

Version: Original Submission **Date:** 4/3/2023

Reviewer name: Tom Madden

Reviewer Comments to Author:

This package runs a pipeline that handles many of the tasks thata user might want to perform on SRA sequences, including dataretrieval, data quality checks, clustering redundant reads, etc.I think the most interesting feature is the ability to search asubset of an SRA run in order to judge it's relevance to a particularproblem.I have some comments/requests.I think the authors need to provide more details about the screeningfeature:In screening mode, 10% of the sequences from a record are downloadedand aligned (by default). I assume this is the first 10% of the reads,as opposed to every 10th read. The authors should state this explicitly.Also, a comment on whether or not looking at only the first 10%might be a non-representative sample would be good.I'm confused about what's being shown in suppl. figures 1 and 2. I thinkthese are alignments for all the sequences in the record, though I'mnot 100% sure. I think the authors would want to show a comparison ofthe prediction that results from sampling 10% of the reads and thefull run. That is, can they demonstrate, even anecdotally, that thisfeature works and is valuable? This is important for readers to takethe article seriously.What is the tRNA database that is used to screen the records for thetwo suppl figures? It seems that should be available to readers and listedin the Data Availability section.How were the 100 SRA runs for the suppl. figures selected? Some weresubmitted by the last author (which I have no issue with), but Ithink it's important that the readers know the basis for the selection.Were they part of another project in the lab, were they chosen based onsome other criteria that made them interesting, etc.?There are issues with the references. References 2 and 9 are the same.7 and 10 are also the same, etc.

Level of Interest

Please indicate how interesting you found the manuscript: Choose an item.

Quality of Written English

Please indicate the quality of language in the manuscript: Choose an item.

Declaration of Competing Interests

Please complete a declaration of competing interests, considering the following questions:

- Have you in the past five years received reimbursements, fees, funding, or salary from an organisation that may in any way gain or lose financially from the publication of this manuscript, either now or in the future?

- Do you hold any stocks or shares in an organisation that may in any way gain or lose financially from the publication of this manuscript, either now or in the future?
- Do you hold or are you currently applying for any patents relating to the content of the manuscript?
- Have you received reimbursements, fees, funding, or salary from an organization that holds or has applied for patents relating to the content of the manuscript?
- Do you have any other financial competing interests?
- Do you have any non-financial competing interests in relation to this paper?

If you can answer no to all of the above, write 'I declare that I have no competing interests' below. If your reply is yes to any, please give details below.

I declare that I have no competing interests.

I agree to the open peer review policy of the journal. I understand that my name will be included on my report to the authors and, if the manuscript is accepted for publication, my named report including any attachments I upload will be posted on the website along with the authors' responses. I agree for my report to be made available under an Open Access Creative Commons CC-BY license (http://creativecommons.org/licenses/by/4.0/). I understand that any comments which I do not wish to be included in my named report can be included as confidential comments to the editors, which will not be published.

Choose an item.

To further support our reviewers, we have joined with Publons, where you can gain additional credit to further highlight your hard work (see: https://publons.com/journal/530/gigascience). On publication of this paper, your review will be automatically added to Publons, you can then choose whether or not to claim your Publons credit. I understand this statement.

Yes Choose an item.
